# Intimate partner violence and negative health consequences: A cross-sectional study among women in a regional sample in Sweden

**DOI:** 10.1177/14034948221148056

**Published:** 2023-01-16

**Authors:** Ylva M.S. Elvin-Nowak, Moa M. Backman-Enelius, Wibke C. Jonas, Julia A. Eriksson, Doris S. Åhlund, Mia M. Barimani

**Affiliations:** 1 Academic Primary Care Centre, Sweden; 2 Department of Women’s and Children’s Health, Karolinska Institutet, Sweden; 3 Division of Biostatistics, Institute of Environmental Medicine, Karolinska Institutet, Sweden; 4 Department of Medical and Health Sciences, Linköping University, Sweden

**Keywords:** Intimate partner violence, violence against women, Sweden, health, cross-sectional

## Abstract

**Background:**

Intimate partner violence (IPV) is a global health problem of enormous proportions. However, little is known about the prevalence or health consequences of IPV among women in Stockholm, Sweden, a city characterised by high levels of gender equality that hosts a large population of people born outside Europe.

**Aims:**

This study aimed to assess the prevalence of exposure to physical, psychological and sexual IPV and its associated background factors and health outcomes.

**Methods:**

This was a cross-sectional study employing a survey containing questions about the previous year’s exposure to IPV that was distributed to 35 midwifery clinics in Stockholm during the autumn of 2020. Any woman who visited any of these midwifery clinics during these two months was eligible to participate.

**Results:**

A total of 2239 women answered the questionnaire, of whom 25.1% reported having been subjected to IPV at some point during their life and 8.7% during the previous year. The most common ongoing exposure was psychological violence, which was reported by 6.6% of the women. Women living with IPV reported poorer self-rated general health and more recurring health symptoms and depression than unexposed women.

**Conclusions:**

**Exposure to IPV is common and is associated with depression, lower general well-being and somatic health problems.**

## Introduction

Intimate partner violence (IPV) includes violence between partners regardless of gender, violence between adult children and their parents and violence from another relative. IPV refers to physical, psychological or sexual violence, including latent violence and coercive control [1]. Within the European Union, 22% of ever-partnered women have experienced physical or sexual violence from a partner [2]. In Sweden, a country with high gender equality and an extensive welfare system, a quarter of women report having been exposed to IPV during their lifetime, of whom 7% have been exposed during the previous 12 months [3].

Several studies have reported long-term negative health consequences of IPV exposure, including chronic pain [4,5], functional gastrointestinal disorders [6], gynaecological issues [7], type 2 diabetes [8] depression, post-traumatic stress disorder (PTSD), anxiety [9] and sleeping disorders [10].

In Sweden, no study has been conducted on the national prevalence of IPV or health outcomes in almost 10 years, and regional statistics from the capital region have not existed until now.

### Aims

This study aimed to assess the prevalence of exposure to physical, psychological and sexual IPV and its associated background factors and health outcomes among women in Stockholm, Sweden.

## Methods

The study had a cross-sectional design and employed a survey containing questions about socio-demographic variables, lifetime and previous-year exposure to IPV and health and lifestyle factors. The focus was ongoing exposure, which, in line with the accepted definition, means exposure during the last 12 months.

### Data collection

During the autumn of 2020 (12 October to 11 December), a survey was carried out at 35 of 64 midwifery clinics in Region Stockholm. The clinics were equally distributed among high- and low-income areas within the region. Any woman who visited any of these clinics during these two months and who could read and write in Swedish, English, Arabic or Somali was eligible to participate. A large proportion of the female population in Stockholm visits a midwifery clinic regularly due to follow-up during pregnancy, cervical screening tests and/or contraceptive counselling (women between 23 and 70 years of age are offered a cervical screening test every three years). In this study, we only included those who were not pregnant and who came to the clinic for cervical screening tests and/or contraceptive counselling. According to the annual report for the midwifery clinics in Stockholm, 48,653 cervical screening tests were conducted and 85,230 physical visits were made for contraceptive counselling in 2020. From the 35 midwifery clinics, 2239 women responded to the survey.

The surveys were distributed by midwives, and the women were asked to complete the questionnaire at the clinics. Due to restrictions related to the COVID-19 pandemic, partners were not allowed to accompany the women during their visits. This ensured the women’s privacy and made the midwifery clinics a safe environment to answer the questionnaire. Questionnaires were returned into a locked box in the waiting room or reception.

### Measures

#### IPV exposure

The questions are based on the Abuse Assessment Screen (AAS), an internationally accepted sensitive and reliable instrument to measure the prevalence of IPV [11]. The original questionnaire was modified to adapt it to the Swedish context, and the options regarding perpetrators were revised: ‘husband’ was replaced by ‘partner’ to include non-married couples and same-sex relationships, and the original inclusion of ‘stranger’ as a reply option was removed to focus solely on violence that occurs in intimate relationships. Questions regarding the frequency of violence were also included in our questionnaire. Women who reported being subjected to abuse either daily, several times a week or several times a month were classified as victims of repeated abuse. Questions regarding psychological violence were added to the questionnaire. Ever-in-life exposure to IPV was measured by asking ‘Have you ever been subjected to psychological or physical violence by your partner or someone else close to you?’ Ongoing exposure to partner violence was based on answers to the following questions: ‘During the past 12 months, have you been punched, kicked, pushed or injured by your partner or someone else close to you?’, ‘During the past 12 months, have you been subjected to psychological violence such as verbal abuse, threats or coercion by your partner or someone else important to you?’ and ‘During the past 12 months, have you been coerced into or subjected to sexual acts against your will by your partner or someone else important to you?’ Women responding affirmatively to at least one of these three questions were defined as having been exposed to IPV during the previous year.

#### Health and lifestyle factors

Mental health status was assessed using the PHQ-9, a validated screening instrument for clinical depression (DSM-5). The PHQ-9 consists of nine items on a four-point scale for measuring impact on daily life over the previous two weeks, with answer options ranging from ‘not at all’ to ‘nearly every day’ [12]. Women were asked to indicate whether they had suffered from any recurring health symptoms during the previous year, such as headaches, migraine, stomach or intestinal problems, eczema, allergy, anxiety, dizziness, gynaecological disorders, pain during sexual intercourse, sleeping problems, and pain in the neck, shoulders or back. The women also had the opportunity to fill in a box for other symptoms; here, the most common symptoms were depression and burnout.

Participants’ health status was classified according to these symptoms. One point was equivalent to one symptom. Multiple answers were allowed. A summary score for all affirmative answers was created, with a maximum score of 12 and minimum of 0. General well-being was assessed by the question ‘How do you rate your general well-being?’, with five alternatives ranging from ‘very good’ to ‘very bad’. For classification of the socio-demographic variables, see Table I.

**Table I.** Exposure to any partner violence during the previous year among 2239 women aged 18–67 years in Stockholm, Sweden, by background characteristics.

	Partner violence		
Characteristic	*n* (%)	Yes, *n* (%)	No, *n* (%)
Age (years)			
<25	476 (20.4)	62 (30.5)	393 (19.2)
25–35	670 (28.7)	63 (31.0)	587 (28.7)
35–44	369 (18.8)	31 (15.3)	320 (15.7)
45–54	235 (10.1)	17 (8.4)	209 (10.2)
⩾55	585 (25.1)	30 (14.8)	534 (26.1)
Education			
Primary school	88 (3.8)	13 (6.5)	67 (3.3)
High school	709 (30.5)	77 (38.3)	596 (29.3)
University	1525 (65.7)	111 (55.2)	1373 (67.4)
Employment			
Employed	1713 (73.6)	140 (69.7)	1515 (74.2)
Unemployed	616 (26.4)	61 (30.3)	526 (25.8)
Living situation			
Husband or partner	1021 (43.8)	54 (26.6)	923 (45.3)
Parents or siblings	260 (11.2)	47 (23.2)	202 (9.9)
Other adults	138 (5.9)	26 (12.8)	104 (5.1)
Children	435 (18.7)	52 (25.6)	370 (18.2)
Alone	475 (20.4)	24 (11.8)	439 (21.5)
Region of birth			
Sweden	1870 (80.2)	157 (77.3)	1659 (81.3)
Europe	204 (8.7)	17 (8.37)	181 (8.9)
Outside Europe	258 (11.1)	29 (14.3)	201 (9.9)

### Ethical considerations

Respondents were informed that participation was voluntary and anonymous and that, by answering the questionnaire, they were giving their informed consent to participate in the study. As the questionnaire contained questions that may cause upset or discomfort, participants were also informed about available helplines and support services. The study was approved by the Swedish Ethical Review Authority (2019-06091).

### Analysis

Associations between background characteristics and IPV were assessed using logistic regression analysis, with statistical significance defined as *p*<0.05. First, three separate regression analyses were performed for each of the four violence variables to examine the connections between ongoing violence and background factors, health variables and lifestyle variables. The model for socio-demographic background factors included age, housing situation, number of children >18 years of age, level of education, employment status and region of origin. The model for health variables included general health conditions, recurring health symptoms, depression and sick leave. The model for lifestyle variables included smoking, alcohol consumption and the use of prescription drugs other than physicians’ prescriptions. All variables that showed significant differences between exposed and unexposed women are included in the final regression models presented in this paper. The results are presented as odds ratios (OR) and 95% confidence intervals (CI). Data analyses were conducted using IBM SPSS Statistics for Windows v26.0 (IBM Corp., Armonk, NY) and R v4.1.0 (The R Foundation for Statistical Computing, Vienna, Austria).

## Results

Exposure to any partner violence during the previous year among 2239 women aged 18–67 years in Stockholm, Sweden, by background characteristics is shown in Table I. Of these, 25.1% reported having been subjected to IPV by a partner or someone else close to them at some point in their life. Altogether, 8.7% stated that they had been subjected to physical, psychological or sexual violence during the previous 12 months (Table II). Psychological violence was the most common form, with 6.6% of all women exposed during the previous year. Physical violence was reported by 3.1% of the women, and sexual violence by 2.6%. Of the women who reported exposure during the previous year, 20.7% had repeatedly been exposed to physical abuse, 37.9% to repeated psychological abuse and 9.4% to repeated sexual violence (Table II).

**Table II.** Frequency and perpetrator among women exposed to relationship violence in the past year and at some point in life (ever in life).

	Last year, physical violence	Last year, psychological violence	Last year, sexual violence	Last year, any violence	Ever, any violence
	*n* (%)	*n* (%)	*n* (%)	*n* (%)	*n* (%)
Total	73 (3.1)	154 (6.6)	61 (2.6)	204 (8.7)	588 (25.1)
Frequency					
Repeatedly	12 (20.7)	54 (37.9)	4 (9.4)	–	–
Occasionally	46 (79)	89 (62)	39 (91)	–	–
Perpetrator					
Partner	22 (35)	49 (36)	19 (38)	–	–
Former partner	26 (41)	51 (38)	12 (24)	–	–
Relative	9 (14)	12 (8.9)	3 (6)	–	–
Other or someone I am currently dating	6 (9.5)	23 (17.5)	16 (32)		

Of the women exposed to physical violence, 41% had been abused by a former partner and 35% by their current partner. Of women subjected to psychological abuse, 38% had been abused by an ex-partner and 36% by their current partner. For sexual violence, 38% had been abused by their present partner, while 32% stated having been abused by ‘other’ or ‘someone I am currently dating’ (Table II).

### Associations between exposure to violence and socio-demographic factors

The prevalence of exposure to violence decreased with age in all four models. Women aged ⩾55 years were at significantly less risk of exposure to any violence during the previous year compared to women aged <25 years (OR=0.48; CI 0.27–0.85; Table III). Similar associations were found when analysing the association between age and physical violence (OR= 0.38; CI 0.15–0.91) and psychological violence (OR= 0.45; CI 0.24–0.84) separately, but not for sexual violence (OR= 0.55; CI 0.18-1.51) (Table IV).

**Table III.** Any form of relationship violence during the previous 12 months.

Characteristic	*n*	OR (95% CI)	*p*
Living situation			
Partner	903	–	–
Parents or siblings***	239	2.56 (1.51–4.34)	<0.001
Other adults***	119	4.50 (2.43–8.21)	<0.001
Children***	390	2.45 (1.57–3.83)	<0.001
Alone	430	1.10 (0.61–1.94)	0.7
Age (years)			
<25	428	–	–
25–34	605	0.71 (0.44–1.15)	0.2
35–44	324	0.89 (0.48–1.63)	0.7
45–54	212	0.61 (0.29–1.22)	0.2
⩾55	512	0.48 (0.27–0.85)	0.012
Highest education			
Primary school	70	–	–
High school	620	0.93 (0.43–2.22)	0.9
University	1391	0.89 (0.41–2.13)	0.8
General health			
Very good	776	–	–
Good	1039	1.27 (0.84–1.93)	0.3
Moderate***	226	2.68 (1.55–4.64)	<0.001
Bad or very bad	40	2.15 (0.82–5.36)	0.11
Number of recurring health symptoms			
0–3	1557	–	–
4–7**	496	1.73 (1.19–2.51)	0.004
8–12**	28	3.66 (1.38–9.22)	0.007
Depression			
None or mild	1820	–	–
Moderate*	176	1.46 (0.88–2.36)	0.13
Severe*	85	1.92 (1.00–3.56)	0.044
Smoking			
No	1831	–	–
Yes, sometimes**	144	2.08 (1.23–3.40)	0.005
Yes, daily**	106	2.35 (1.31–4.09)	0.003

*** *p*<0.001; **p<0.01; *p<0.05.

**Table IV.** Physical, psychological and sexual violence respectively during the previous 12 months.

Characteristic	*n*	OR (95% CI)	*p*
*Physical violence*			
Living situation			
Partner	989	–	–
Parents or siblings	258	1.94 (0.85–4.38)	0.11
Other adults	133	5.10 (2.23–11.5)	<0.001
Children	425	2.80 (1.43–5.53)	0.003
Alone	469	0.96 (0.37–2.33)	>0.9
Age (years)			–
<25	466	–	–
25–34	650	0.69 (0.35–1.39)	0.3
35–44	359	0.93 (0.38–2.23)	0.9
45–54	230	0.91 (0.34–2.29)	0.8
⩾55		0.38 (0.15–0.91)	0.035
Region of birth			
Sweden	1,836	–	–
Europe	201	0.79(0.23–2.00)	0.7
Outside Europe	237	2.16(1.10–3.97)	0.018
Number of recurring health symptoms			
0–3	1,709	–	–
4–7	534	1.53 (0.88–2.58)	0.12
8–12	31	7.55 (2.55–19.5)	<0.001
Smoking			
No	1999	–	–
Yes, sometimes	156	2.37 (1.15–4.56)	0.014
Yes, daily	119	2.31 (0.97–4.91)	0.041
*Psychological violence*			
Living situation			
Partner	914	–	–
Parents or siblings	241	2.35 (1.30–4.23)	0.005
Other adults	121	5.37 (2.78–10.2)	<0.001
Children	398	2.39 (1.44–3.96)	<0.001
Alone	433	1.24 (0.64–2.34)	0.5
Age (years)			
<25	430	–	–
25–34	612	0.63 (0.38–1.07)	0.086
35–44	329	0.71 (0.35–1.39)	0.3
45–54	217	0.64 (0.29–1.34)	0.3
⩾55	519	0.45 (0.24–0.84)	0.013
General health			
Very good	780	–	–
Good	1058	1.58 (0.99–2.57)	0.061
Moderate	227	2.68 (1.43–5.03)	0.002
Bad or very bad	42	2.17 (0.74–5.87)	0.14
Symptoms of depression			
None or mild	1840	–	–
Moderate	179	1.20 (0.68–2.05)	0.5
Severe	88	1.55 (0.75–3.06)	0.2
Number of recurring health symptoms			
0–3	1574	–	–
4–7	505	2.02 (1.35–3.03)	<0.001
8–12	28	2.75 (0.87–7.71)	0.066
Smoking			
No	1855	–	–
Yes, sometimes	145	2.31 (1.3–-3.90)	0.002
Yes, daily	107	3.06 (1.69–5.34)	<0.001
*Sexual violence*			
Living situation			
Partner	965	–	–
Parents or siblings	251	7.27 (3.19–17.6)	<0.001
Other adults	127	3.72 (1.08–11.4)	0.026
Children	417	3.62 (1.67–8.30)	0.001
Alone	457	0.80 (0.21–2.58)	0.7
Age (years)			
<25	458	–	–
25–34	643	1.70 (0.85–3.49)	0.14
35–44	347	1.26 (0.41–3.54)	0.7
45–54	223	0.48 (0.07–1.96)	0.4
⩾55	546	0.55 (0.18–1.51)	0.3
General health			
Very good	812	–	–
Good	1117	0.99 (0.50–1.98)	>0.9
Moderate	242	3.11 (1.45–6.70)	0.003
Bad or very bad Medication use^a^	46	5.54 (1.71–16.0)	0.002
No	2081	–	–
Yes, sometimes	42	2.74 (0.72–8.06)	0.095
Yes, daily	94	2.09 (0.74–5.04)	0.13
Smoking			
No	1946	–	–
Yes, sometimes	154	2.26 (1.04–4.57)	0.029
Yes, daily	117	1.55 (0.59–3.59)	0.3

a Use of prescription medicines for purposes other than prescribed.

Women cohabiting with a parent or sibling (OR=2.56; CI 1.51–4.34) or another adult (OR=4.50; CI 2.43–8.21) or those living alone with children (OR=2.45; CI 1.57–3.83) were significantly more likely to report exposure to any violence during the previous year compared to women cohabiting with a husband or partner (Table III). These associations were also found for psychological violence and sexual violence when analysed separately, whilst only cohabitation with other adults (*p*<0.001) or with children (*p*=0.003) were risk factors for physical violence (Table IV). Women born outside Europe reported significantly greater exposure to physical violence than women born in Sweden (OR=2.16; CI 1.10–3.97; Table IV). The fully adjusted models found no significant connection between exposure to violence and education or employment status (data not shown).

### Associations between exposure to violence and health and lifestyle factors

Participants reporting exposure to any form of physical, psychological or sexual violence during the previous year were more likely to suffer from severe depression (OR=1.92; CI 1.00–3.56; Table III). Exposed women were also more likely to self-rate well-being as moderate compared to the reference group ‘very good’ (OR=2.68; CI 1.55–4.64; Table III). The connection between poorer self-rated well-being and exposure to violence was particularly strong for sexual violence: women who had been exposed to sexual violence were significantly more likely to report their overall health status as poor or very poor (OR=5.54; CI 1.71–16.0; Table IV).

The results showed a significant association between more recurring health symptoms and exposure to violence during the previous year. Women who reported between four and seven recurring health symptoms were more likely to have experienced any violence (OR=1.73; CI 1.19–2.51; Table III) compared to the reference group, and for the women who reported between 8 and 12 recurring health symptoms, the OR was 3.66 (CI 1.38–9.22; Table III). The association between exposure to violence and a larger number of recurring health symptoms was also found for physical violence and psychological violence (Table IV) when analysed separately, but not for sexual violence (data not shown).

Women who smoked were more likely to report any partner violence, either daily (OR=2.35; CI 1.31–4.09; Table III) or occasionally (OR=2.08; CI 1.23–3.40; Table III) than women who didn’t smoke. The connection between daily smoking and exposure to violence was particularly strong for psychological violence (OR=3.06; CI 1.69–5.34; Table IV). The fully adjusted models found no significant connection between exposure to violence and alcohol consumption, or the use of prescription medicines for purposes other than prescribed by a medical doctor (data not shown).

## Discussion

The results of this regional survey, which included a large sample of women, demonstrate that almost 9% of the participating women were exposed to ongoing IPV. In line with previous research [3,9], psychological violence was the most common form of violence in this study. For these women, everyday life might be characterised by humiliation, coercive control, criticism and threats of physical violence [9]. Our results show that many women lived with repeated violence from a partner or former partner. Long-term exposure to IPV often leads to normalisation in terms of habituation to the violence and a tendency to hide it [13]. Since women exposed to IPV consume a lot of health care [14] and exhibit many different symptoms [15], recognition of this group of women is extremely important for health-care staff.

In agreement with earlier studies, the present study shows a strong association between exposure to IPV and recurring negative health symptoms in terms of pain in different parts of the body [4,5], gynaecological problems [7] and anxiety and depression [9]. Women exposed to any kind of IPV also assessed their self-rated health as lower compared to non-exposed women. Our results are in alignment with one study indicating sexual violence to be extremely harmful [16].

Our findings are in line with several other studies and show that young age is one of the most central risk factors for IPV [17,18]. We also found that victimised women were more likely to be living with their parents, siblings or other adults. This may be explained by the fact that younger women are more likely to be living at home or in shared houses due to their age. Exposed women were also more likely to be living alone with children, a finding that is also in line with one Norwegian study [7]. These cases may be examples of continuing post-separation assault after leaving a violent relationship.

Our findings also showed that women born outside Europe had an increased risk of being exposed to physical IPV compared to women born in Sweden. This finding is in line with two other Swedish studies [19,20]. Higher prevalence rates of violence among foreign-born women have often been attributed to lower socio-economic status [19]. However, our findings could not confirm this. A possible explanation for the greater degree of exposure to physical violence among women born outside Europe may be connected to honour-related violence, which is a problem that has received increasing attention in Sweden [21].

In agreement with previous research, we found that women who smoked cigarettes were more likely to be exposed to violence [22]. Smoking may function as a coping mechanism for exposure to violence and serve to reduce symptoms of anxiety temporarily [23].

### Strengths and limitations

A strength of the study was that data collection was carried out at both smaller and larger midwifery clinics throughout the region and spread across different socio-demographic areas. The surveys were also available in several languages, which is likely to have made it easier for women who do not usually participate in surveys to do so. The surveys were distributed in midwifery clinics, an environment that women generally perceive as safe. The fact that the survey was anonymous and that no partners were allowed to accompany them during their visit due to the COVID-19 pandemic probably made it easier for abused women to answer the survey.

A limitation of this study is that it has not been possible to make a non-response analysis based on the design we have chosen. Thus, we do not know how many eligible women did not respond, which may affect generalisability. The design, involving a survey distributed by midwives and not accounting for the number of questionnaires distributed, was chosen on the basis that we wanted to burden these midwives as little as possible. However, we think that we have reached an adequate number of women according to our data collection. Another factor that also affected generalisability is that the women in our study were more highly educated (73%) than the population of Region Stockholm women in general (59%). Although women from 114 nationalities were represented in our data, the percentage of women who were foreign born and responded to the survey (20%) was lower than for the region (26%). Finally, our study had a cross-sectional design and therefore our results should be interpreted with caution in terms of causal relationships between IPV and health factors.

## Conclusions

This study shows that almost 9% of the respondents are currently living with IPV. Women exposed to IPV have extensive health problems and have many contacts with health care. Therefore, IPV is a key issue for the health-care system to address. However, knowledge about the association between IPV and health problems and illness is often limited among medical staff. In turn, people living with IPV tend not to disclose their exposure to IPV. Increasing awareness about the magnitude of the problem and the association between IPV and decreased general well-being, different somatic problems and depressive symptoms may be the first step towards better care for women living with IPV.
